# Psychological Distress in Patients Treated for Renal Cell Carcinoma: A Systematic Literature Review

**DOI:** 10.3390/jcm11216383

**Published:** 2022-10-28

**Authors:** Liliana Vartolomei, Manuela Schmidinger, Mihai Dorin Vartolomei, Shahrokh F. Shariat

**Affiliations:** 1 Department of Urology, Medical University of Vienna, 1090 Vienna, Austria; 2 Institution Organizing University Doctoral Studies I.O.S.U.D, George Emil Palade University of Medicine, Pharmacy, Sciences and Technology from Târgu Mureș, 540139 Târgu Mureș, Romania; 3 Department of Urology & Comprehensive Cancer Center, Clinical Division of Oncology, Medical University of Vienna, 1090 Vienna, Austria; 4 Institute for Urology and Reproductive Health, I.M. Sechenov First Moscow State Medical University, Moscow 119435, Russia; 5 Department of Urology, Weill Cornell Medical College, New York, NY 1300, USA; 6 Department of Urology, University of Texas Southwestern, Dallas, TX 75390, USA; 7 Karl Landsteiner Institute of Urology and Andrology, 1090 Vienna, Austria; 8 Department of Urology, Second Faculty of Medicine, Charles University, 110 00 Prague, Czech Republic; 9 Hourani Center for Applied Scientific Research, Al Ahlizza Amman University, Amman 19328, Jordan

**Keywords:** nephrectomy, renal cancer, depression, anxiety, metastasis, survival, psychological distress

## Abstract

**(1)** **Background:** The incidence of psychological distress and its impact on renal cell carcinoma (RCC) patients is unclear. Our aim was to analyze the literature regarding the prevalence of psychological distress and its impact on patients with non-metastatic or metastatic RCC; **(2) Methods:** A systematic search of five databases was performed. Studies were considered eligible if they included patients with RCC, had a prospective or retrospective design, and assessed anxiety, depression, or psychological distress at any time during treatment or follow-up. Exclusion criteria: no treatment for RCC, or not providing data for RCC patients; **(3) Results:** A total of 15 studies were included. Reported psychological distress was up to 77% and the prevalence of depressive and anxiety symptoms were up to 77.6% and 68.3% in patients with non-metastatic RCC. There was no association of depression with overall survival (OS) in patients with non-metastatic RCC treated by radical nephrectomy; on the contrary, in patients with metastatic disease, depression had an impact on OS. Limitations are related to the quality of the included studies; **(4) Conclusions**: Patients with RCC reported a high level of psychological distress like other cancer patients. It seems that for patients with localized disease, psychological distress does not impact OS, while it does in those with metastatic disease.

## 1. Introduction

Renal cell carcinoma (RCC) represents almost half of the newly diagnosed urinary tract cancers and has an estimated incidence in the US alone of 79,000 in 2022 [1] and a worldwide **reported** incidence of more than 431,288 [2]. Treatment of RCC may vary from active surveillance, ablative therapies, partial or radical nephrectomy for localized disease until cytoreductive nephrectomy and systemic treatment (targeted therapies and/or immunotherapy) for metastatic disease [3]. 

Psychological distress refers to non-specific symptoms that include stress, anxiety and depression; increased levels of psychological distress may indicate the beginning of serious conditions such as major depressive disorder, anxiety disorder, schizophrenia, somatization disorder, or a variety of other clinical conditions [4]. **In practice, it is often seen that many patients deny any symptoms of psychological distress [5,6] and often they self-treat with ethanol [7] and/or social recreational agents [8].**

Cancer patients with psychological distress (symptoms of depression and anxiety) seem to have higher cancer-specific and overall mortality. Indeed, according to a recent meta-analysis, clinical depression and anxiety significantly increased the risk of cancer incidence (adjusted RR: 1.13, 95%CI: 1.06–1.19), cancer-specific mortality (1.21, 95%CI 1.16–1.26), and all-cause mortality (1.24, 95%CI 1.13–1.35) in cancer patients [9].

Depression and anxiety incidences were reported to be high in patients with urological cancers: 70% in patients with bladder cancer [10], 17% in prostate cancer [11] and 20% in testicle cancer [12]. Both overall and disease-specific mortality outcomes have been shown to be lower in bladder and prostate cancer patients affected by psychological distress [13]. Nevertheless, there is a scarcity of research regarding systematic data related to the impact of psychological distress, depression and/or anxiety on oncological outcomes among RCC patients.

To fill this void, we performed a systematic review to analyze the existing literature regarding the incidence of psychological distress, depression and/or anxiety and their impact on oncological outcomes in patients with non-metastatic and metastatic RCC separately.

## 2. Materials and Methods

**Figure 1:**
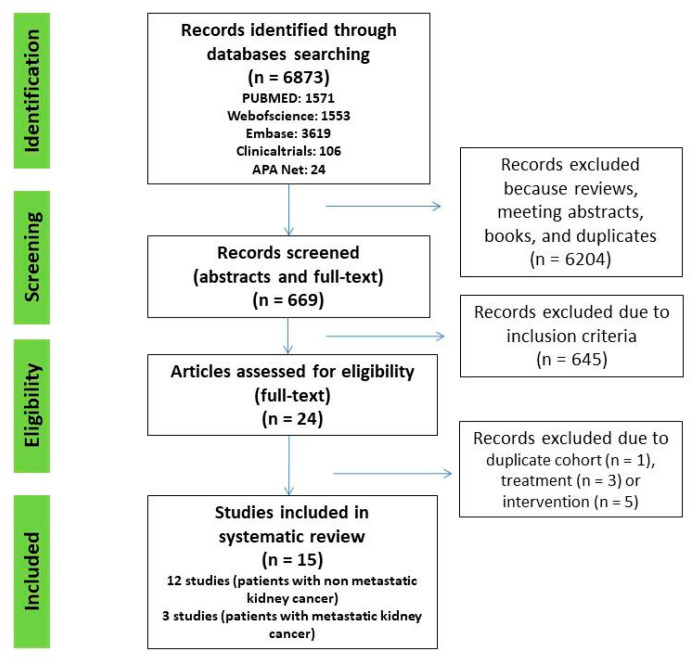
PRISMA flow chart of study selection process.

A systematic search of Web of Science, PubMed, Embase, Clinicaltrials.gov, and American Psychological Association (APA) PsycNet was performed on 1 April 2022, using any combination of the terms: anxiety (EXP) OR depression (EXP) OR psychological distress (EXP) AND nephrectomy (EXP) OR renal carcinoma (EXP) OR kidney cancer (EXP). All original articles that fulfilled the inclusion criteria were included. Additional crosschecking of reference lists and hand search to find additional studies was also performed in Google Scholar (Figure 1). 

### 2.1. Protocol 

The protocol of this systematic review followed the Cochrane handbook [14] and the Preferred Reporting Items for Systematic Reviews and Meta-analysis (PRISMA) criteria (www.prisma-statement.org (accessed on 30 August 2022)) [15]. The present systematic review was registered in the PROSPERO International prospective register of systematic reviews (registration no. CRD42021282104).

### 2.2. Inclusion and Exclusion Criteria 

The PICOS (Population, Intervention, Comparator, Outcome and Study design) approach was utilized to define study eligibility. Initial studies were considered eligible if they reported psychological distress (P) before nephrectomy for renal cancer (I) and reported psychological distress after nephrectomy at specific time points during follow-up (C) to determine if there is a change in psychological distress status (O), using validated scales (S). However, only one study met this criteria [16], so we decided to expand the inclusion criteria to studies that included patients with kidney cancer, had a prospective or retrospective design, and assessed anxiety, depression and/or psychological distress at any time during treatment and/or follow-up. Exclusion criteria: studies that included patients undergoing no treatment for RCC, or not providing data on anxiety, depression and/or psychological distress separately for RCC patients. 

The primary outcome was the prevalence of anxiety, depression and/or psychological distress among patients with non-metastatic and metastatic RCC separately. The secondary outcome was the impact of anxiety, depression and/or psychological distress on outcomes of patients with non-metastatic and metastatic RCC, separately. 

For each selected study, the following items were recorded independently by two investigators (L.V and M.D.V): first author’s name, year of publication, country, design, number of patients, treatment option, patients’ characteristics, questionnaires used, reported results (primary outcome: prevalence of anxiety, depression and/or psychological distress and secondary outcome: impact on oncologic outcomes) and follow-up. Two investigators (L.V and M.D.V) independently manually conducted a literature search and extracted data from the included full-text articles; disagreements were resolved by consensus.

### 2.3. Study Quality and Risk of Bias Evaluation

Study quality was determined by the Newcastle-Ottawa Scale (NOS) [17] for cohort studies. Thresholds for converting the NOS were established according to the Agency for Health Research and Quality (AHRQ) standards. Five studies had good quality, one study had fair quality and the other nine had poor quality (Supplementary Material Table S1A).

The “risk-of-bias” (RoB) evaluation of each study was assessed according to the Cochrane Handbook for systematic reviews of interventions for including non-randomized studies. RoB was determined by examining the risk of pre-assigned confounders. The main confounding factors were age, gender, marital status, socioeconomic status, and occupation. The presence of confounders was determined by consensus (Supplementary Material Table S1B).

## 3. Results

A total of 6873 abstracts and titles were initially identified in all five databases. After removal of reviews, meeting abstracts, books, and duplicates, 669 remained. Then, 645 articles were excluded after screening titles and abstracts. We assessed 24 full text articles and, finally, 15 studies were included (Figure 1). 

Ten studies assessed the prevalence of psychological distress, anxiety and/or depression in non-metastatic RCC (Table 1), two the association of psychological distress, anxiety and/or depression in non-metastatic RCC with survivals outcomes (Table 2) and another two the association of psychological distress, anxiety and/or depression in metastatic RCC with survivals outcomes; one reported changes in anxiety and depression levels at 12 weeks after treatment in metastatic RCC patients (Table 3).

The RoB evaluation showed that two studies had a *’low risk of bias’* (in green) and 13 studies had a *’high risk of bias’* (in red, see Supplementary Material Table S1B). All included studies reported results considering age and gender as cofounders and five studies reported results considering all main confounding factors.

### 3.1. Psychological Distress, Anxiety and/or Depression in Patients with Non-Metastatic Renal Cell Carcinoma

Ten studies that assessed psychological distress, anxiety and/or depression in non-metastatic RCC patient were identified [16,18,19,20,21,22,23,24,25,26]. They included a total of 2188 patients (841 females, 38.4%) from different countries such as Turkey [18], Canada [19], Korea [20], the United States (US) [16,21,24,25] (849 patients, 38.8%), Germany [22], China [23] and Italy [26]. These studies used different validated tools to assess psychological distress, anxiety and/or depression such as the Edmonton Symptom Assessment System–revised (ESAS-r), SF-12 Health Survey, Perceived Stress Scale (PSS), Center for Epidemiologic Studies Depression Scale (CES-D), Zung Self-Rating Anxiety Scale (Zung SAS), Patient Health Questionnaire-9, National Comprehensive Cancer Network (NCCN) Distress Thermometer, Beck Depression Inventory–II (BDI-II), State–Trait Anxiety Inventory (STAI), General Health Questionnaire (GHQ), Hospital Anxiety and Depression Scale (HADS), as well as the Social Problem Questionnaire (SPQ.). Despite the variety of scales used, the psychological distress rate was reported to be as high as 77% [21] and the prevalence of depressive and anxiety symptoms was up to 77.6% and 68.3% [23], respectively. Nevertheless, these studies investigated and reported distress, anxiety and/or depression at different stages of disease such as pre-nephrectomy or during follow-up, with only one study reporting baseline data and at 24 weeks [16]. Time-dependent reporting found no change in depression nor in anxiety scores (Table 1). The heterogeneity among studies prohibited the performance of a meta-analysis of the available data.

### 3.2. Impact of Anxiety and/or Depression on Oncological Outcomes in Patients with Non-Metastatic Renal Cell Carcinoma

Two studies reported oncological outcomes in relation to anxiety and depression in patients with non-metastatic RCC. One included 1990 patients from the US [27] and one 182 patients from China [28]. When analyzing a retrospective database, Packiam et al. [27] found no association of anxiety or depression with recurrence, distant metastases, overall survival (OS) or death from RCC after 10 years of follow-up. On the contrary, Song et al. [28] found that patients with sustained anxiety according to the Hospital Anxiety and Depression Scale–Anxiety (HADS-A) were more likely to die from any cause with a median follow-up of 43 months. Even in the context of a randomized clinical trial (RCT), other oncological outcomes such as recurrence, metastases or cancer specific survival were not analyzed. Both studies failed to detect an association of depression with OS (Table 2).

### 3.3. Impact of Psychological Distress, Anxiety and/or Depression on Patients with Metastatic Renal Cell Carcinoma

Three studies investigated the impact of psychological distress, anxiety and/or depression, on patients with metastatic RCC. Two studies included 304 patients from the US [29,30] and one included 127 patients from China [31]. Different instruments were used to measure distress, anxiety and/or depression including a touch screen-based instrument— either HADS or CES-D. A meta-analysis was not performed due to heterogeneity between studies. Cohen et al. [30] found a statistically significant association between clinical depression measured with CES-D (score ≥ 16), and OS with a hazard ratio (HR) of 1.5 (95%CI 1.00–2.23). Similarly, in the study of Bergerot et al. [29], a poorer OS was observed in patients with high distress compared to those with low distress (20 mo. vs. 45.8 mo.), but statistical significance was not reached. In a prospective evaluation of a Chinese cohort, Wang et al. [31] reported that, in patients with metastatic RCC, anxiety and depression increased 3 months after interferon-α treatment (Table 3).

**Table 3:** Studies that assessed the impact of psychological distress, anxiety and/or depression on patients with metastatic renal cell carcinoma (RCC).

No.	First Author	Year	Country	Study Design	No. Patients	Patients Characteristics	Questionnaires	Results	Follow-Up
1	Bergerot et al.	2019 [29]	United States of America	retrospective	102 29 females (28.4%)	age ≥ 18 years, had histologically confirmed RCC, and had radiographic evidence of metastatic disease.	A touch screen–based instrument was used to assess biopsychosocial problem-related distress. The instrument surveyed 22 core items on a 5-point Likert scale ranging from 1 (not a problem) to 5 (very severe problem). Items rated ≥3 were considered to reflect high distress.	The median OS was 43.7 months (95% confidence interval [95%CI] = 35.5, 52.5) for the overall cohort; 20.0 months (95%CI = 16.0, 55.9) in patients with high distress, and 45.8 months (95%CI = 36.1, 55.5) in those with low distress (*p* = 0.81).	n.a
2	Wang et al.	2018 [31]	China	prospective	127 26 females (20.4%)	(I) diagnosed as mRCC according to clinical, imaging and pathological findings; (II) age above 18 years; (III) about to receive IFN-α treatment; (IV) life expectancy longer than 12 months; (V) able to be followed up regularly and complete the assessment questionnaires. Patients were excluded if they (I) had brain metastasis; (II) accompanied with other solid tumors or hematological malignance; (III) had uncontrolled hypertension, severe infection or primary organ failure; (IV) were pregnant or lactating, or planned for pregnancy	HADS-Anxiety score, HADS-Depression score and EORTC QLQ-C30 Scale at baseline and at 12 weeks	The percentages of anxiety and depression both increased at W12 compared with W0. Depression (43.3% vs. 31.5%, *p* = 0.004) and anxiety (32.3% vs. 22%, *p* = 0.035)	12 weeks
3	Cohen et al.	2012 [30]	United States of America	prospective	202 46 females (23%)	newly diagnosed metastatic RCC, a life expectancy of greater than 4 months, a Zubrod performance status of less than or equal to 2, and no serious intercurrent illnesses.	(Centers for Epidemiologic Studies—Depression; SF-36 Health Status Survey; Duke Social Support Index; Coping Operations Preference Enquiry	CES-D scores (*p* = 0.05, HR = 1.5, 95%CI for HR: 1.00–2.23) were predictors for decrease survival	1.8 years

Legend: OS: overall survival; HADS: Hospital Anxiety and Depression Scale.

## 4. Discussion

A high level of distress, depression, and/or anxiety in patients with non-metastatic RCC was reported. This is in line with other serious urological neoplasia such as bladder cancer [32]. A prevalence of clinical severe depression in up to 12 % of patients was reported [18], which is much higher than the reported World Health Organization (WHO) rate of 5.7% for depression in the elderly [33] or in times of COVID-19 (28% for depression; 26.9% for anxiety; 24.1% for post-traumatic stress symptoms; 36.5% for stress; 50% for psychological distress) in the general population. As a limitation, many studies included within prevalence rate patients with mild, moderate or severe depressive or anxiety symptoms, or did not stratify patients according to symptom severity [34]. Even within the included studies in the present review, there is no clear delimitation regarding the presence of clinically significant symptoms of depression, anxiety, or psychological distress.

Another meta-analysis reported an even lower probable prevalence during the COVID-19 pandemic for anxiety (20.7%, 95%CI 12.9–29.7), depression (18.1%, 95%CI 13.0–23.9), and psychological distress (13.0%, 95%CI 0–34.1) [35]. Due to delays in surgeries, the COVID-19 pandemic might have also influenced psychological distress, depression and/or anxiety among patients with localized RCC; as a result, patients with T1a RCC had a significantly worse cancer-specific survival rate (HR 1.67, 95%CI 1.23–2.27, *p* < 0.01) [36].

However, when analyzing the data regarding the impact of distress, depression, or anxiety on oncological outcomes in patients with non-metastatic RCC, it seems that these conditions have no influence on mid- and long-term outcomes. This may be related to the good overall prognosis of non-metastatic RCC [37] and probably in these settings it would be interesting to evaluate the impact on quality of life (QoL) and further adherence to follow-up.

Conversely, in metastatic disease, we found that a high level of distress and depression is associated with OS; other endpoints were not analyzed, such as recurrence, progression, or cancer-specific survival. This correlation was also shown in both bladder and prostate cancers [13]. It is of the utmost importance to identify the patients at risk and to treat them according to their symptom severity. If these symptoms are severe, then these conditions may influence treatment adherence as was shown in other cancers [38,39]. Moreover, a large multinational survey showed that 50% of RCC patients are ‘very often’ or ‘always’ experiencing disease-related anxiety [40].

The present systematic review analyzed the up-to-date data regarding the prevalence and the impact of psychological distress, anxiety and/or depression, on patients with non-metastatic or metastatic RCC. However, some limitations should be pointed out such as the poor quality of the data, risk of bias, lack of randomized clinical studies and standardization of the reported results that prevailed when performing a meta-analysis.

## 5. Conclusions

Patients with RCC reported a high level of psychological distress similar to that of patients with other urological malignancies. It seems that for patients with localized disease, these symptoms had no impact on oncological outcomes. Conversely, in metastatic disease, psychological distress is associated with worse outcomes. The impact of psychological distress on therapy adherence and long-term wellbeing warrants further research. Providers, patients, and families need awareness and targeted interventions to identify and help affected patients in a sensitive and effective manner.

## Figures and Tables

**Table 1:** Studies that assessed psychological distress, anxiety and/or depression in patients with non-metastatic renal cell carcinoma (RCC).

No.	First Author	Year	Country	Study Design	No. Patients	Patients Characteristics	Treatment	Questionnaires	Results	Follow-Up
1	Demirtaş et al.	2021 [18]	Turkey	Cross-sectional	250 (66 females 27%)	Kidney cancer	surgery had 102 patients (40.8%) type of surgery n.a	Hospital Depression and Anxiety Scale (HADS), and Perceived Stress Scale (PSS)	HADS-Anxiety symptoms in the study were found in 91.2% patients; 98 (39.2%) participants had mild, 99 (39.6%) had moderate, 31 (12.4%) had severe anxiety symptoms. HADS-Depression symptoms were found in 87.2% patients; 87 (34.8%) participants had mild, 101 (40.4%) had moderate, 30 (12%) had severe depression symptoms.	None Evaluation some time (n.a) after treatment
2	Ajaj et al.	2020 [19]	Canada	Retrospective 2014–2017	495 (184 females 37.2%)	Consecutive patients diagnosed with non-metastatic RCC	PN 93 females (62.8%) vs. 148 males (61.7%)	The Edmonton Symptom Assessment System—revised (ESAS-r)	Increasing age was shown to be associated with a lower Psychological distress sub-score (PDSS) score after diagnosis [B = −0.135, 95%CI −0.238 to (−0.032), *p* = 0.011] and after nephrectomy [B = −0.078, 95%CI −0.139 to (−0.018), *p* = 0.012]. PDSS was significantly higher in females after diagnosis (8.5 vs. 5.1, *p* = 0.018), after biopsy (8.9 vs. 4.1, *p* = 0.003), and after surgery (6.5 vs. 4.4, *p* = 0.007), while there was no difference at the last follow-up (5.9 vs. 5, *p* = 0.379).	None Evaluation at 37 mo. for females and 26 mo. for males after treatment
3	Shin et al.	2019 [20]	Korea	Case-control between April 2014 and December 2015	108 (31 females 28.7%)	non-metastatic RCC	n.a	European Organization for Research and Treatment of Cancer QLQ-C30, the Duke-UNC Functional Social Support Questionnaire and the Patient Health Questionnaire-9.	At 2 years no statistically significant difference was observed in terms of depressive symptoms between kidney cancer survivors and the general population.	None Evaluation at least 1 year after surgery
4	Bergerot et al.	2019 [21]	USA	Online survey from 1 April to 15 June 2017	450 (224 females 56%)	non-metastatic RCC (74%)	n.a	NCCN Distress Thermometer (NCCN-DT)	77 % of patients reported moderate-to-severe distress. Distress was significantly associated with female gender, younger age, non-clear cell histology and presence of recurrence	None Evaluation some time (n.a) after treatment
5	Draeger et al.	2018 [22]	Germany	Cross-sectional	74 (20 females 27%)	Consecutive RCC patients irrespective of tumor stage (localized versus advanced) 71 were N0M0	71 RN	[NCCN Distress Thermometer (NCCN-DT), Hornheider Screening Instrument (HSI)]	The main identified stressors were anxiety (28%), pain (27%), nervousness (26%), sadness (20%), sorrow (20%) and sleep difficulties (20%)	None Evaluation at time of treatment
6	Li et al.	2016 [23]	China	Cross-sectional from July 2013 to July 2014	268 (100 females 37.3%)	Kidney cancer stage I-III (mostly non-metastatic RCC)	n.a	Center for Epidemiologic Studies Depression Scale (CES-D), Zung Self-Rating Anxiety Scale, Resilience Scale-14, and Perceived Stress Scale-10	The prevalence of depressive and anxiety symptoms was 77.6% and 68.3% in renal cancer patients.	None Evaluation one week after surgery
7	Thekdi et al.	2016 [24]	USA	Baseline data of a randomized controlled trial evaluating the benefits of an expressive writing intervention on quality of life outcomes	287 (118 females 42%)	Kidney cancer Stage I-IV (mostly non-metastatic RCC)	Surgery 195 (70.3)	CES-D, Impact of Events Scale (IES), MD Anderson Symptom Inventory (MDASI), Brief Fatigue Inventory (BFI) and Pittsburgh Sleep Quality Index (PSQI)	15.2% were identified as having comorbid (Post-traumatic Stress Symptoms) PTSS and depressive symptoms; 24.1% PTSS alone; 5.9% depressive symptoms alone	None Evaluation before treatment
8	Ames et al.	2011 [16]	USA	Prospective between October 2005 and January 2008	28 (8 females 29%)	localized RCC	RN	The Functional Assessment of Cancer Therapy–General (FACT-G), Medical Outcomes Study 36-item short form survey (SF-36), The Profile of Mood States–Brief (POMS-B), Beck Depression Inventory–II (BDI-II), State-Trait Anxiety Inventory (STAI) prior to nephrectomy and at 4, 12, and 24 weeks post-nephrectomy and completion of individual semi-structured interviews 4 weeks post-nephrectomy	No significant change was observed in BDI –II scores: 5.5 (4.8) baseline vs. 8.7 (9.7) at 24 weeks, *p* = 0.081 No significant change was observed in STAI scores at baseline 34.9 (12.9) vs. 32.1 (8.9) at 24 weeks, *p* = 0.25	Evaluation at baseline and at 24 weeks after treatment
9	Anastasiadis et al.	2003 [25]	USA	Cross-sectional survey of a random sample of patients	84 (42 females 50%)	Kidney cancer	RN (up to 90%)	Watts Sexual Function Questionnaire (WSFQ), the SF-12 Health Survey, CES-D, and the Revised Dyadic Adjustment Scale	51% of men and 57% of women reported depressive symptoms (CES-D > 16) at more than 3 years after diagnosis	None Evaluation at 3.2 years for females and 3.7 years for males after treatment
10	Ficarra et al.	2002 [26]	Italy	Cross-sectional	144 (48 females 33.3%)	T1N0M0 RCC	56 patients (39%) have been treated with elective NSS and 88 (61%) underwent RN	General Health Questionnaire (GHQ), HADS, Social Problem Questionnaire (SPQ.)	Higher scores for anxiety 2.77 ± 2.77 vs. 1.79 ± 2.47, *p* = 0.003 and depression 2.08 ± 2.32 vs. 1.70 ± 2.80 *p* = 0.015 were reported in the RN group compared to those that underwent NSS	None Evaluation at 55 ± 36 months after treatment

Legend PN: partial nephrectomy, RN: radical nephrectomy, NCCN: National Comprehensive Cancer Network.

**Table 2:** Studies that assessed the impact of anxiety or depression on oncological outcomes of patients with non-metastatic renal cell carcinoma (RCC).

No.	First Author	Year	Country	Study Design	No. Patients	Patients Characteristics	Treatment	Questionnaires	Results	Follow-Up
1	Packiam et al.	2020 [27]	USA	Retrospective between 1995 and 2011	1990 (701 females 35%)	non-metastatic RCC	RN 1144 (57%)	Baseline anxiety and depression were identified using ICD-9 Codes (197 patients with anxiety and/or depression)	No significant differences were noted regarding local ipsilateral recurrence, distant metastases, overall survival or death from RCC between patients with vs. without anxiety or depression.	10 years
2	Song et al.	2018 [28]	China	Randomized clinical trial	182	RCC patients were randomly allocated 1:1 in two groups intensive patients’ care program IPCP group (IPCP plus usual care) or a control group (only usual care) (I) diagnosed as RCC according to clinical, imaging and pathological findings; (II) age above 18 years; (III) unilateral renal cell carcinoma; (IV) received radical nephrectomy; (V) able to complete the questionnaire of assessments.	RN	Hospital Anxiety and Depression Scale anxiety/ depression (HADS-A/HADS-D) Zung Self Rating Anxiety/Depression Scale (SAS/SDS) Evaluation at baseline and at 12 months after treatment	Patients with sustained anxiety assessed by the HADS-A score had worse OS compared with that of patients without sustained anxiety assessed by HADS-A score (*p* = 0.026). Patients with sustained anxiety assessed by SDS score also had a shorter OS compared with patients without sustained anxiety assessed by SAS score (*p* = 0.012). No difference of OS between patients with or without depression assessed by HADS-D score (*p* = 0.166) or SDS score (*p* = 0.131)	43 mo.

Legend: RN: radical nephrectomy; ICD-9: International Classification of Diseases,9th Revision; OS: overall survival.

## Data Availability

Not applicable.

## Supplementary Materials

The following supporting information can be downloaded at: https://www.mdpi.com/article/10.3390/jcm11216383/s1, Table S1: (A) Newcastle-Ottawa Scale of the eligible studies and (B) Risk of bias summary of the eligible studies.

## References

[1] Siegel R.L., Miller K.D., Fuchs H.E., Jemal A. (2022). Cancer Statistics, 2022. CA Cancer J. Clin..

[2] Sung H., Ferlay J., Siegel R.L., Laversanne M., Soerjomataram I., Jemal A., Bray F. (2021). Global Cancer Statistics 2020: GLOBOCAN Estimates of Incidence and Mortality Worldwide for 36 Cancers in 185 Countries. CA Cancer J. Clin..

[3] Ljungberg B., Albiges L., Abu-Ghanem Y., Bedke J., Capitanio U., Dabestani S., Fernández-Pello S., Giles R.H., Hofmann F., Hora M. (2022). European Association of Urology Guidelines on Renal Cell Carcinoma: The 2022 Update. Eur. Urol..

[4] Cuijpers P., Smits N., Donker T., ten Have M., de Graaf R. (2009). Screening for Mood and Anxiety Disorders with the Five-Item, the Three-Item, and the Two-Item Mental Health Inventory. Psychiatry Res..

[5] Livingston J.D., Youssef G.J., Francis L.M., Greenwood C.J., Olsson C.A., Macdonald J.A. (2021). Hidden in Plain Sight? Men’s Coping Patterns and Psychological Distress Before and During the COVID-19 Pandemic. Front. Psychiatry.

[6] Ho C.S.H., Chua J., Tay G.W.N. (2022). The Diagnostic and Predictive Potential of Personality Traits and Coping Styles in Major Depressive Disorder. BMC Psychiatry.

[7] Mc Hugh R., McBride O. (2022). Investigating the Nature of Depressive Experiences in Adults Who Self-Medicate Low Mood with Alcohol. Alcohol Fayettev. N.

[8] Asselin A., Lamarre O.B., Chamberland R., McNeil S.-J., Demers E., Zongo A. (2022). A Description of Self-Medication with Cannabis among Adults with Legal Access to Cannabis in Quebec, Canada. J. Cannabis Res..

[9] Wang Y.-H., Li J.-Q., Shi J.-F., Que J.-Y., Liu J.-J., Lappin J.M., Leung J., Ravindran A.V., Chen W.-Q., Qiao Y.-L. (2020). Depression and Anxiety in Relation to Cancer Incidence and Mortality: A Systematic Review and Meta-Analysis of Cohort Studies. Mol. Psychiatry.

[10] Vartolomei L., Ferro M., Mirone V., Shariat S.F., Vartolomei M.D. (2018). Systematic Review: Depression and Anxiety Prevalence in Bladder Cancer Patients. Bladder Cancer Amst. Neth..

[11] Brunckhorst O., Hashemi S., Martin A., George G., Van Hemelrijck M., Dasgupta P., Stewart R., Ahmed K. (2021). Depression, Anxiety, and Suicidality in Patients with Prostate Cancer: A Systematic Review and Meta-Analysis of Observational Studies. Prostate Cancer Prostatic Dis..

[12] Smith A.B., Butow P., Olver I., Luckett T., Grimison P., Toner G.C., Stockler M.R., Hovey E., Stubbs J., Turner S. (2016). The Prevalence, Severity, and Correlates of Psychological Distress and Impaired Health-Related Quality of Life Following Treatment for Testicular Cancer: A Survivorship Study. J. Cancer Surviv. Res. Pract..

[13] Dinesh A.A., Helena Pagani Soares Pinto S., Brunckhorst O., Dasgupta P., Ahmed K. (2021). Anxiety, Depression and Urological Cancer Outcomes: A Systematic Review. Urol. Oncol..

[14] Cochrane Handbook for Systematic Reviews of Interventions. http://handbook-5-1.cochrane.org/.

[15] Moher D., Liberati A., Tetzlaff J., Altman D.G. (2010). PRISMA Group Preferred Reporting Items for Systematic Reviews and Meta-Analyses: The PRISMA Statement. Int. J. Surg. Lond. Engl..

[16] Ames S.C., Parker A.S., Crook J.E., Diehl N.N., Tan W.W., Williams C.R., Ames G.E. (2011). Quality of Life of Patients Undergoing Surgical Treatment for Newly-Diagnosed, Clinically Localized Renal Cell Carcinoma. J. Psychosoc. Oncol..

[17] Wells G.A., Shea B., O’Connell D., Peterson J., Welch V., Losos M., Tugwell P. The Newcastle-Ottawa Scale (NOS) for Assessing the Quality If Nonrandomized Studies in Meta-Analyses. http://www.ohri.ca/programs/clinical_epidemiology/oxford.htm.

[18] Demirtaş T., Temircan Z. (2022). Examining the Relationship between Depression, Anxiety and Stress in Kidney Cancer Patients. J. Kidney Cancer VHL.

[19] Ajaj R., Cáceres J.O.H., Berlin A., Wallis C.J.D., Chandrasekar T., Klaassen Z., Ahmad A.E., Leao R., Finelli A., Fleshner N. (2020). Gender-Based Psychological and Physical Distress Differences in Patients Diagnosed with Non-Metastatic Renal Cell Carcinoma. World J. Urol..

[20] Shin D.W., Park H.S., Lee S.H., Jeon S.H., Cho S., Kang S.H., Park S.C., Park J.H., Park J. (2019). Health-Related Quality of Life, Perceived Social Support, and Depression in Disease-Free Survivors Who Underwent Curative Surgery Only for Prostate, Kidney and Bladder Cancer: Comparison among Survivors and with the General Population. Cancer Res. Treat..

[21] Bergerot C.D., Battle D., Staehler M.D., Pal S.K. (2019). Distress in Patients with Renal Cell Carcinoma: A Curious Gap in Knowledge. BJU Int..

[22] Draeger D.L., Sievert K.-D., Hakenberg O.W. (2018). Analysis of Psychosocial Stress Factors in Patients with Renal Cancer. Ther. Adv. Urol..

[23] Li M., Wang L. (2016). The Associations of Psychological Stress with Depressive and Anxiety Symptoms among Chinese Bladder and Renal Cancer Patients: The Mediating Role of Resilience. PLoS ONE.

[24] Thekdi S.M., Milbury K., Spelman A., Wei Q., Wood C., Matin S.F., Tannir N., Jonasch E., Pisters L., Cohen L. (2015). Posttraumatic Stress and Depressive Symptoms in Renal Cell Carcinoma: Association with Quality of Life and Utility of Single-Item Distress Screening. Psychooncology.

[25] Anastasiadis A.G., Davis A.R., Sawczuk I.S., Fleming M., Perelman M.A., Burchardt M., Shabsigh R. (2003). Quality of Life Aspects in Kidney Cancer Patients: Data from a National Registry. Support. Care Cancer Off. J. Multinatl. Assoc. Support. Care Cancer.

[26] Ficarra V., Novella G., Sarti A., Novara G., Galfano A., Cavalleri S., Artibani W. (2002). Psycho-Social Well-Being and General Health Status after Surgical Treatment for Localized Renal Cell Carcinoma. Int. Urol. Nephrol..

[27] Packiam V.T., Tyson Ii M.D., Tsivian M., Lohse C.M., Boorjian S.A., Cheville J.C., Costello B.A., Leibovich B.C., Thompson R.H. (2020). The Association of Anxiety and Depression with Perioperative and Oncologic Outcomes among Patients with Clear Cell Renal Cell Carcinoma Undergoing Nephrectomy. Urol. Oncol..

[28] Song B., Zhang Y., Wang Y., An X. (2018). Intensive Patients’ Care Program Ameliorates Anxiety and Depression, and Sustained Anxiety Correlates with Worse Overall Survival in Renal Cell Carcinoma Patients Underwent Radical Nephrectomy. Transl. Cancer Res..

[29] Bergerot C.D., Clark K.L., Ashing K.T., Bergerot P.G., Obenchain R., Dizman N., Hsu J., Philip E., Loscalzo M., Pal S.K. (2019). Biopsychosocial Distress and Clinical Outcome in Metastatic Renal Cell Carcinoma. Palliat. Support. Care.

[30] Cohen L., Cole S.W., Sood A.K., Prinsloo S., Kirschbaum C., Arevalo J.M.G., Jennings N.B., Scott S., Vence L., Wei Q. (2012). Depressive Symptoms and Cortisol Rhythmicity Predict Survival in Patients with Renal Cell Carcinoma: Role of Inflammatory Signaling. PLoS ONE.

[31] Wang Y., Song B., Zhang Y., Li H. (2018). Evaluation and Predictive Factors Analyses for Patient-Self-Reported Depression, Anxiety and Quality of Life in Patients with Metastatic Renal Cell Carcinoma Underwent Interferon-α Treatment: A Prospective Cohort Study. Transl. Cancer Res..

[32] Vartolomei L., Vartolomei M.D., Shariat S.F. (2020). Bladder Cancer: Depression, Anxiety, and Suicidality among the Highest-Risk Oncology Patients. Eur. Urol. Focus.

[33] Institute of Health Metrics and Evaluation Global Health Data Exchange (GHDx). http://Ghdx.Healthdata.Org/Gbd-Results-Tool?Params=gbd-Api-2019-Permalink/D780dffbe8a381b25e1416884959e88b.

[34] Nochaiwong S., Ruengorn C., Thavorn K., Hutton B., Awiphan R., Phosuya C., Ruanta Y., Wongpakaran N., Wongpakaran T. (2021). Global Prevalence of Mental Health Issues among the General Population during the Coronavirus Disease-2019 Pandemic: A Systematic Review and Meta-Analysis. Sci. Rep..

[35] Leung C.M.C., Ho M.K., Bharwani A.A., Cogo-Moreira H., Wang Y., Chow M.S.C., Fan X., Galea S., Leung G.M., Ni M.Y. (2022). Mental Disorders Following COVID-19 and Other Epidemics: A Systematic Review and Meta-Analysis. Transl. Psychiatry.

[36] Chan V.W.-S., Tan W.S., Leow J.J., Tan W.P., Ong W.L.K., Chiu P.K.-F., Gurung P., Pirola G.M., Orecchia L., Liew M.P.C. (2021). Delayed Surgery for Localised and Metastatic Renal Cell Carcinoma: A Systematic Review and Meta-Analysis for the COVID-19 Pandemic. World J. Urol..

[37] Palumbo C., Mistretta F.A., Knipper S., Pecoraro A., Tian Z., Shariat S.F., Saad F., Simeone C., Briganti A., Antonelli A. (2020). Conditional Survival of Patients With Nonmetastatic Renal Cell Carcinoma: How Cancer-Specific Mortality Changes After Nephrectomy. J. Natl. Compr. Cancer Netw..

[38] Yussof I., Mohd Tahir N.A., Hatah E., Mohamed Shah N. (2022). Factors Influencing Five-Year Adherence to Adjuvant Endocrine Therapy in Breast Cancer Patients: A Systematic Review. Breast Edinb. Scotl..

[39] Kim S.J., Kang D., Park Y., Mun Y.-C., Kim K., Kim J.S., Min C.-K., Cho J. (2021). Impact of Depression on Adherence to Lenalidomide plus Low-Dose Dexamethasone in Patients with Relapsed or Refractory Myeloma. Support. Care Cancer Off. J. Multinatl. Assoc. Support. Care Cancer.

[40] Giles R.H., Marconi L., Martinez R., Maskens D., Kastrati K., Castro C., Julian Mauro J.C., Bick R., Heng D.Y.C., Larkin J. (2022). Patient-Reported Experience of Diagnosis, Management, and Burden of Renal Cell Carcinomas: Results >2,000 Patients in 41 Countries, with Focus on Older Patients. J. Clin. Oncol..

