# Supplementary material for: Psychological Distress in Patients Treated for Renal Cell Carcinoma: A Systematic Literature Review
Source: J Clin Med. 2022 Oct 28;11(21):6383. doi: 10.3390/jcm11216383 (PMC9657659; doi:10.3390/jcm11216383)
Supplement: Supplementary file 1 [file jcm-11-06383-s001.zip › jcm-1971025-supplementary.pdf]

Supplementary Table S1: (A) Newcastle-Ottawa Scale of the eligible studies and (B) Risk of bias summary of the eligible studies.

A.

Study and year	Selection	Comparability	Exposure	Total
Demirtaş et al. 2021[18]	★★		★	**3**
Ajaj et al. 2020[19]	★★	★	★★	**5**
Shin et al. 2019[20]	★★★★	★	★★	**7**
Bergerot et al. 2019[21]	★★		★	**3**
Draeger et al. 2018[22]	★★		★	**3**
Li et al. 2016[23]	★★		★	**3**
Thekdi et al. 2016[24]	★★		★	**3**
Ames et al. 2011[16]	★★★		★★	**5**
Anastasiadis et al. 2003[25]	★★★		★	**4**
Ficarra et al. 2002[26]				
Packiam et al. 2020[27]				
Song et al. 2018[28]				
Bergerot et al. 2019[29]				
Wang et al.				

2018[31]				
Cohen et al. 2012[30]				

B.

	Random sequence generation (selection bias)	Allocation concealment (selection bias)	Blinding of participants (performance bias)	Blinding of outcome (detection bias)	Incomplete outcome data (attrition bias)	Selective reporting (reporting bias)	NOS	Adjusted for age	Adjusted for gender	Adjusted for marital status	Adjusted for socioeconomic status	Adjusted for occupation
**Study and year**	**Assessment of the main biases**							**Analysis of cofounders**				
Demirtaş et al. 2021[18]							**3**					
Ajaj et al. 2020[19]							**5**					
Shin et al. 2019[20]							**7**					
Bergerot et al. 2019[21]							**3**					
Draeger et al. 2018[22]							**3**					
Li et al. 2016[23]							**3**					
Thekdi et al. 2016[24]							**3**					
Ames et al. 2011[16]							**5**					
Anastasiadis et al. 2003[25]							**4**					

Ficarra et al. 2002[26]												
Packiam et al. 2020[27]												
Song et al. 2018[28]												
Bergerot et al. 2019[29]												
Wang et al. 2018[31]												
Cohen et al. 2012[30]												
